# Morphological characteristics of ocular toxoplasmosis and its regression pattern on swept-source optical coherence tomography angiography: a case report

**DOI:** 10.1186/s12886-019-1209-8

**Published:** 2019-09-05

**Authors:** Joong Hyun Park, Sang-Yoon Lee, Eun Kyoung Lee

**Affiliations:** 1 Department of Ophthalmology, Jeju National University School of Medicine, Jeju National University Hospital, Jeju, Republic of Korea; 2 Department of Ophthalmology, Seoul National University School of Medicine, Seoul National University Hospital, #101, Daehak-ro, Jongno-gu, Seoul, Republic of Korea

**Keywords:** Case report, Ocular toxoplasmosis, Morphological changes, Swept-source optical coherence tomography angiography

## Abstract

**Background:**

To report the successful treatment of ocular toxoplasmosis and present the use of multimodal imaging to describe the changes in ocular toxoplasmic lesions subsequent to treatment.

**Case presentation:**

A 73-year-old female visited the clinic with decreased visual acuity in the left eye. Fundus examination showed severe vitreous haze with yellow-white infiltrates near the foveal center. Spectral-domain optical coherence tomography (SD-OCT) revealed disorganization of the retinal structure with markedly thickened choroid beneath the active lesion. Highly elevated serum titers of IgG antibodies against *Toxoplasma gondii* were observed. Topical and systemic steroids with oral Bactrim were administered after a diagnosis of ocular toxoplasmosis was made. After improvement in the severity of vitritis, structural en face swept-source optical coherence tomography (SS-OCT) imaging demonstrated diffuse choroidal dilation with many collateral vascular branches surrounding the active lesion. Eight intravitreal injections of clindamycin (1 mg/0.1 ml) were administered at 1- to 2-week intervals along with systemic antibiotics and steroids. After the treatment, the toxoplasmic lesion resolved to an atrophic chorioretinal scar. Dilated choroidal vessel size was normalized and collateral vascular branches were markedly constricted on structural en face SS-OCT images.

**Conclusions:**

This is the first detailed report on the morphological changes in the choroidal vasculature surrounding ocular toxoplasmic lesions that were characterized using SS-OCT-A imaging. Multimodal imaging with SS-OCT-A can be valuable in clinical diagnosis as well as in clarifying the mechanism of choroidal structural changes in ocular toxoplasmosis.

## Background

Ocular toxoplasmosis is one of the most common cause of posterior uveitis caused by an intracellular parasite, *Toxoplasma gondii* [1, 2]. The infection may be acquired by consumption of raw meat containing cysts or ingestion of water or food contaminated by oocytes [3, 4]. Following invasion of the parasite into the eye, the tachyzoite remains latent in the cyst under the control of the immune response of host. In event of trigger of rupture, the tachyzoite is converted to bradyzoite, and the inflammatory response is activated [5, 6]. The retina is most frequently affected, and choroid, vitreous, and anterior chamber can be also involved. Active inflammation tends to regress over few months with scar formation, and reactivation adjacent to old scar lesion is commonly observed in ocular toxoplasmosis [7]. The diagnosis of ocular toxoplasmosis is based on the clinical findings; however, various imaging modalities can be helpful in arriving at an accurate diagnosis and developing a successful treatment plan.

The introduction of spectral-domain optical coherence tomography (SD-OCT) [8, 9] allowed for layer-by-layer evaluation of the retina, including good visualization of the photoreceptor microstructures. Furthermore, with the introduction of enhanced depth imaging [10] and swept-source optical coherence tomography (SS-OCT) [11, 12], visualization of the choroid has become possible. More recently, a new imaging modality, optical coherence tomography angiography (OCT-A) [13], was developed, which provides a clear visualization of the retinal and choroidal microvasculature even without injection of contrast dye.

Several studies have demonstrated the vitreal and retinal morphological characteristics in toxoplasmic lesions using SD-OCT [5, 14]. However, detailed morphological changes of the choroidal vasculature occurring from the active phase of the disease to remission after treatment using swept-source OCT-A (SS-OCT-A) have not yet been investigated. The present report describes a case of ocular toxoplasmosis that was successfully treated and presents the use of multimodal imaging to evaluate the changes in toxoplasmic lesions subsequent to treatment.

## Case presentation

**Fig. 1:**
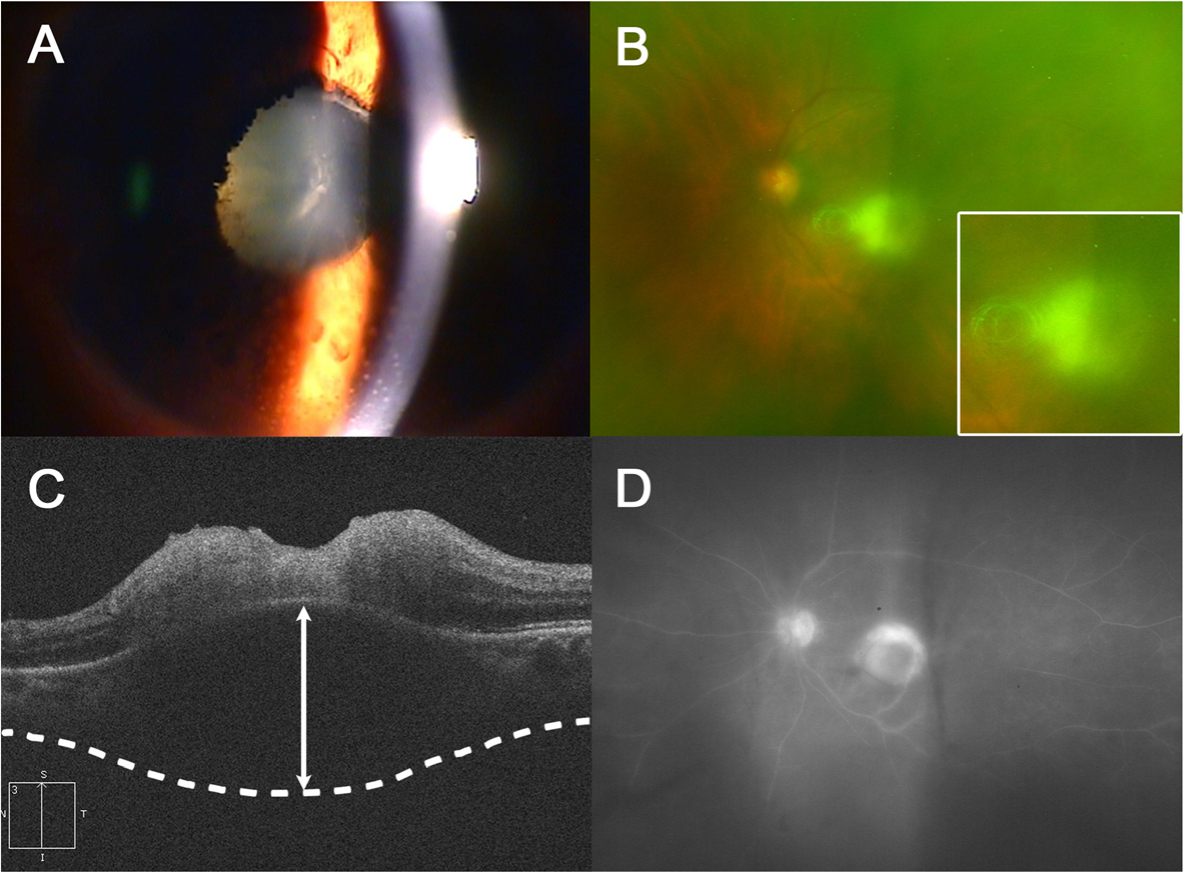
Multi-modal imaging at initial presentation. **a**. Anterior segment photograph revealing mutton-fat keratic precipitates and peripupillary fibrous membrane with posterior synechiae. **b**. Wide-field fundus photography demonstrating macular yellow-white infiltrates with vitreous haze (white frame, magnified image). **c**. Disorganization of the retinal structure with markedly thickened choroid (white double-headed arrow) as observed on SD-OCT. **d**. Wide-field fluorescein angiography showing optic disc leakage and hyperfluorescence of the active lesion

A 73-year-old female presented with decreased visual acuity of her left eye that lasted for a month. The patient was systemically healthy and did not report a history of diabetes or hypertension. Her best-corrected visual acuity was 20/20 in the right eye and counting fingers at 30 cm in the left eye. The intraocular pressure was 14 mmHg in the right eye and 13 mmHg in the left eye. Slit-lamp examination showed numerous mutton-fat keratic precipitates, 2+ anterior chamber cells, moderate cortical and nuclear sclerosis cataract, and fibrinous membrane bridging the pupil, with 360 degrees of posterior synechiae (Fig. 1a). Fundus examination revealed prominent vitreous haze along with yellow-white infiltrates near the foveal center (Fig. 1b). The SD-OCT demonstrated hyperreflectivity of the intraretinal layers with disorganization of retinal structure and retinal pigment epithelium (RPE) elevation. Moreover, remarkably thickened choroid beneath the active lesion with hyporeflectivity of the choroid was also noted (Fig. 1c). Wide-field fluorescein angiography showed hyperfluorescence of the active lesion and leakage of dye from the optic disc (Fig. 1d). Findings in the right eye were nonspecific with the exception of mild cataract.

The patient indicated consumption of raw pork prior to her current illness. On laboratory examination, the titer of serum IgG antibodies against *Toxoplasma gondii* was found to be > 650.0 IU/ml (normal < 1.0 IU/ml) and titer of serum IgG antibodies against *Toxocara canis* was 2.062 (normal < 1.140). Based on the clinical features and laboratory findings, a diagnosis of ocular toxoplasmosis of the left eye was made. The patient was treated with oral Bactrim (80 mg trimethoprim + 400 mg sulfamethoxazole) 2 tablets twice daily, oral prednisolone 50 mg daily, topical prednisolone acetate 1.0% (Pred-forte®) every 2 h, and 2.0% homatropine twice daily.

**Fig. 2:**
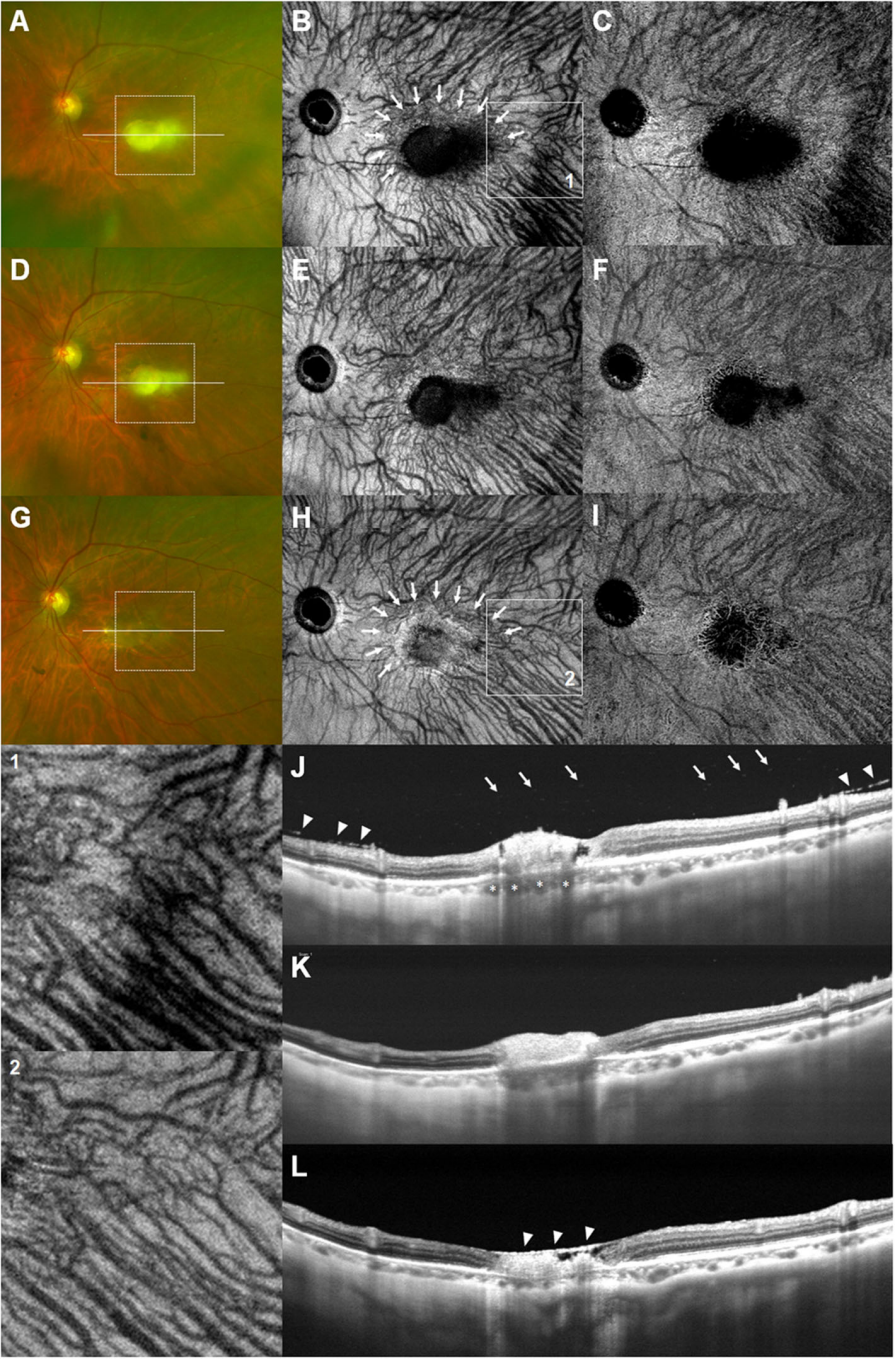
Fundus photographs (**a**, **d**, **g**), structural en face SS-OCT images (**b**, **e**, **h**), SS-OCT-A images (**c**, **f**, **i**), and SS-OCT B-scans (**j**, **k**, **l**) during the course of treatment. At 4 weeks after treatment, **a**. Vitreous haze decreased, but yellow-white infiltrate remained near the foveal center. **b**. Many collateral vascular branches surrounding the lesion (white arrows) and diffuse choroidal dilation, which is more remarkable in magnified image (Box 1) are shown. **c**. SS-OCT-A image showed congested choroidal vasculature. **d**, **e**, **f**. After two intravitreal injections of clindamycin, the size of macular infiltrates decreased with more discrete margins. **g**. After 3.5 months of additional treatment, the macular lesion changed to chorioretinal scar. **h**, **i**. Normalization of dilated choroidal vessels and constriction of collateral branch vessels around the lesion (white arrows), which is more remarkable in magnified image (Box 2) are seen. **j**. An SS-OCT B-scan through the lesion (white line on the fundus photograph) demonstrated disruption of the neurosensory retina, interruption of the photoreceptor inner and outer segment junction and RPE elevation. Multiple hyperreflective dots in the vitreous cavity indicating severe vitritis (white arrows), thickened posterior hyaloid (white arrowheads), and dilated Haller’s layer vessels (white asterisks) were also noted. **k**. The choroidal thickness decreased further. **l**. Atrophic retinal thinning, overlying thickened posterior hyaloid (white arrowheads), and pronounced choroidal thinning are seen

After 4 weeks, slit-lamp examination showed reduced inflammatory reaction in the anterior chamber. The grade of anterior chamber cells decreased to 1+ and posterior synechiae were broken. The dose of oral prednisolone was tapered gradually over 4 weeks while the course of oral Bactrim was continued. Fundus examination revealed decrease in vitreous opacities; however, the yellow-white infiltrates persisted near the foveal center (Fig. 2a). The SS-OCT-A device (PLEX Elite 9000; Carl Zeiss Meditec, Inc., Dublin, CA) was used to evaluate the morphological characteristics of the active lesions. Structural en face SS-OCT imaging at the level of the choroid 150 μm below the RPE revealed hyporeflectivity of the macular lesion with diffuse choroidal dilation and many collateral vascular branches surrounding the lesion (Fig. 2b), which is more remarkable in magnified image (indicated by Box 1). SS-OCT-A image at a level deeper to choriocapillaris demonstrated congested choroidal vasculature (Fig. 2c). The SS-OCT B-scan showed disruption of the neurosensory retinal layers with interruption of the photoreceptor inner and outer segment junction and RPE elevation. Multiple hyperreflective dots in the vitreous cavity indicating severe vitritis and dilated Haller’s layer vessels were also noted (Fig. 2j). The choroid beneath the lesions remained thick and hyporefective; however, a decrease in the thickness was observed when compared to the thickness recorded in the first visit.

Due to the proximity of the lesion to the macula area, the decision to combine intravitreal injections with systemic antibiotic therapy was made. Therefore, two intravitreal injections of clindamycin (1 mg/0.1 ml) were administered at weekly intervals, along with systemic antibiotics and corticosteroids. Follow up after two weeks revealed diminished vitreous opacity and reduction in the size of the macular lesion with more discrete margins (Fig. 2d, e, and f). Thinning of the hyperreflective lesion of the intraretinal layers and further reduction in the choroidal thickness was observed (Fig. 2k). Subsequently, the patient was administered six additional intravitreal injections of clindamycin (1 mg/0.1 ml) at an interval of 1 to 2 weeks. Oral Bactrim was discontinued after 8 weeks and the dose of oral prednisolone was tapered gradually over the next four months.

Four months after the first visit, the best-corrected visual acuity of the patient was 20/125 and the intraocular pressure was 15 mmHg in the left eye. Slit-lamp examination showed trace anterior chamber cells with the absence of keratic precipitates. Fundus examination revealed presence of an atrophic scar in the macular area (Fig. 2g). Structural en face SS-OCT imaging demonstrated normalization of congested and dilated choroidal vessels (Fig. 2h), which is more remarkable in magnified images (indicated by Box 2). Marked constriction of the collateral vascular branches around the chorioretinal scar was observed. SS-OCT-A image revealed that the choroidal vasculature are visible within the atrophic macular lesion (Fig. 2i). The SS-OCT B-scan showed thinning and disorganization of the neurosensory retina with the presence of overlying thickened posterior hyaloid (Fig. 2l). The thickness of the choroid beneath the lesions decreased to a level below the normal value. The choriocapillaries, Sattler’s layer, and Haller’s layer disappeared partially and became hyperreflective. At the final follow up, 9 months after the first visit, only topical prednisolone acetate 1.0% was being continued and the patient had no recurrence of the condition.

## Discussion and conclusion

Ocular toxoplasmosis frequently presents as a focal necrotizing retinochoroiditis. Necrosis of the retina and choroid with destruction of the surrounding tissues is found within the active lesion. In addition, the condition is commonly associated with intense vitreous inflammation. “Headlight in the fog” appearance refers to a bright white reflex that is seen when the light of the indirect ophthalmoscope is shone onto the back of the eye, as seen in cases of severe vitritis [15]. Active lesions demonstrate a fluffy, yellow-white focus of retinitis. With the introduction of SD-OCT, disorganization and hyperreflectivity of the neurosensory retinal layers were observed in active toxoplasmic lesions [14]. Interestingly, substantial thickening and hyporeflectivity of the choroid beneath the active lesions were found [14]. However, detailed morphological changes of the choroidal vasculature in ocular toxoplasmosis have not been investigated. To the best of our knowledge, this is the first study to demonstrate the morphological characteristics of the choroidal layers in toxoplasmic lesions based on en face SS-OCT and SS-OCT-A imaging.

In the present study, the en face OCT images at the level of the medium and large choroidal vessels visualized the temporal structural changes with regard to treatment response. Changes in the en face SS-OCT images may be considered as a secondary change due to signal attenuation rather than vascular remodeling. However, the magnified images of en face SS-OCT and OCT B-scans showed more clearly that dilated choroidal vasculature became constricted after treatment. In the active phase of the disease, diffuse choroidal dilation and many collateral vascular branches surrounding the lesion were observed. The exact cause of choroidal thickening cannot be ascertained without histological confirmation. However, it is reasonable to speculate that choroidal thickening may be caused by inflammatory cell infiltration and increase in blood flow [16]. Previous studies have reported that choroidal thickening occurs during the acute phase of active intraocular inflammation, whereas choroidal thinning occurs during the remission phase of the disease. Other inflammatory conditions associated with choroidal thickening include Bechet’s disease [17], Vogt-Koyanagi-Harada (VKH) disease [18], or sympathetic ophthalmia [19]. A histopathology study of acute VKH disease revealed that thickening of the choroid was due to diffuse infiltration of lymphocytes, macrophages, and epithelioid cells [20]. More recently, a study demonstrated that changes in submacular choroidal thickness occur even when the active retinochoroidal toxoplasmic lesion lies outside the macula [21]. The changes observed in the present study support the observations of previous studies in that the inflammation caused by ocular toxoplasmosis affects not only the active lesion but also wide areas of choroidal structures. The post-treatment en face OCT imaging revealed normalization of the congested and dilated choroidal vessels and marked constriction of the collateral vascular branches around the lesion. These findings indicated attenuation of acute inflammation with treatment. The results of the present study suggest that choroidal evaluation using en face SS-OCT and SS-OCT-A is useful for monitoring disease activity and may contribute substantially to therapeutic decision making in patients with ocular toxoplasmosis.

The aim of the treatment was to arrest multiplication of the parasite during the active stage and to minimize damage to the intraocular tissues [22, 23]. The classical chemotherapeutic regimen for toxoplasmic retinochoroitidis consists of pyrimethamine and sulfadiazine, plus corticosteroids. However, this classical treatment may be associated with significant adverse effects, along with challenges in patient compliance due to the high number of daily oral medications. There was a trial to make a combination with less adverse side effects and good compliance [24]. A combination of Trimethoprim/sulfamethoxazole plus oral prednisolone has been suggested as an alternative treatment option with similar efficacy in terms of reduction in size of the retinal lesion and improvement in vision when compared with the classical therapy [24]. Another therapeutic option suggested in the literature is injection of intravitreal clindamycin and dexamethasone [25]. A randomized clinical trial reported that intravitreal injection of clindamycin and dexamethasone may be an acceptable alternative to the classical therapy [26]. In the present study, the patient suffered from sight-threatening toxoplasmic retinochoroiditis; therefore, the treatment comprised of systemic antibiotic therapy plus oral prednisolone along with intravitreal injection of clindamycin, resulting in a favorable outcome. Therapeutic strategy for ocular toxoplasmosis should be individualized based on the compliance of the patient, location, characteristics of the lesion, and disease activity.

In conclusion, multimodal imaging with SS-OCT and OCT-A was useful in evaluation of retinal and choroidal morphological characteristics in active toxoplasmic retinochoroiditis. In particular, en face SS-OCT images appears to be a valuable modality to elucidate the mechanism of structural changes in the choroid in patients with ocular toxoplsmosis.

## Data Availability

All the data supporting the conclusions of this article are included within the article and its figures.

## Abbreviations

OCT-A: optical coherence tomography angiography
RPE: retinal pigment epithelium
SD-OCT: spectral-domain optical coherence tomography
SS-OCT: swept-source optical coherence tomography
SS-OCT-A: swept-source optical coherence tomography angiography
VKH: Vogt-Koyanagi-Harada
